# Metabolic landscape in venous thrombosis: insights into molecular biology and therapeutic implications

**DOI:** 10.1080/07853890.2024.2401112

**Published:** 2024-09-19

**Authors:** Zheng Cao, Xuejun Jiang, Yiyu He, Xiaoxin Zheng

**Affiliations:** a Department of Cardiology, Renmin Hospital of Wuhan University, Wuhan, Hubei, China; b Cardiovascular Research Institute, Wuhan University, Wuhan, Hubei, China; c Hubei Key Laboratory of Cardiology, Renmin Hospital of Wuhan University, Wuhan, Hubei, China

**Keywords:** Venous thrombosis, metabolism, inflammatory, oxidative stress

## Abstract

The findings of the last decade suggest a complex link between inflammatory cells, coagulation, and the activation of platelets and their synergistic interaction to promote venous thrombosis. Inflammation is present throughout the process of venous thrombosis, and various metabolic pathways of erythrocytes, endothelial cells, and immune cells involved in venous thrombosis, including glucose metabolism, lipid metabolism, homocysteine metabolism, and oxidative stress, are associated with inflammation. While the metabolic microenvironment has been identified as a marker of malignancy, recent studies have revealed that for cancer thrombosis, alterations in the metabolic microenvironment appear to also be a potential risk. In this review, we discuss how the synergy between metabolism and thrombosis drives thrombotic disease. We also explore the great potential of anti-inflammatory strategies targeting venous thrombosis and the complex link between anti-inflammation and metabolism. Furthermore, we suggest how we can use our existing knowledge to reduce the risk of venous thrombosis.

## Introduction

Deep venous thrombosis (DVT) and pulmonary embolism (PE) make up venous thromboembolism (VTE). According to the current global epidemiological statistics, the incidence of VTE varies widely by age, from 0.1 per 1,000 in adolescence to 8 per 1,000 in individuals in their eighties [1]. In the United States, at least 200,000 patients are diagnosed with PE each year [2,3], and approximately 100,000 to 180,000 people die from VTE each year [4]. Risk factors for lower extremity VTE are mostly acquired, including tumors, inflammation, prolonged braking, obesity, and antiphospholipid syndrome [5–8], differing from arterial thrombosis in that hyperlipidemia, hypertension, and diabetes [9]. The pathological mechanism of venous thrombosis is determined by the Virchow triad, including blood stasis, blood hypercoagulability, and vascular endothelial activation [10,11]. Numerous studies have shown the involvement of innate immune cells, such as neutrophils and mast cells, in the formation and propagation of venous thrombosis [12,13], and eosinophils seem to have less influence on the formation of venous thrombosis. In contrast, endothelial and immune cells have unique biochemical and histological features, such as oxidative stress, abnormal cellular metabolism, and mitochondrial abnormalities [14]. These may lead to abnormalities in their metabolic microenvironment, which in turn promote the formation of venous thrombosis. Current research on venous thrombosis has focused on the relationship between inflammation and thrombosis, in contrast to cellular metabolism, which has focused on oxidative stress and cellular autophagy. Cellular metabolism and products may promote inflammation, leading to cellular damage and oxidative stress, resulting in the promotion of thrombosis.

Anticoagulation is still the cornerstone of VTE treatment. Anti-inflammatory therapy might be not only effective in reducing the recurrence rate of VTE but could also be a proper way to avoid one of the more problematic aspects of anticoagulation – bleeding [15,16]. In addition to neutrophil extracellular traps (NETs), several more promising therapeutic targets are currently recognized, including Krüppel-like factor 2 and high mobility group protein-1 [17]. However, studies on the effects of cellular metabolism and the metabolic microenvironment on VTE formation are lacking. In this regard, we reviewed and outlined the impact of cellular metabolic status on VTE formation and the link to these two therapeutic targets to provide further insights into preventing and treating VTE.

## Link between venous thrombosis and inflammation

**Figure 1.**
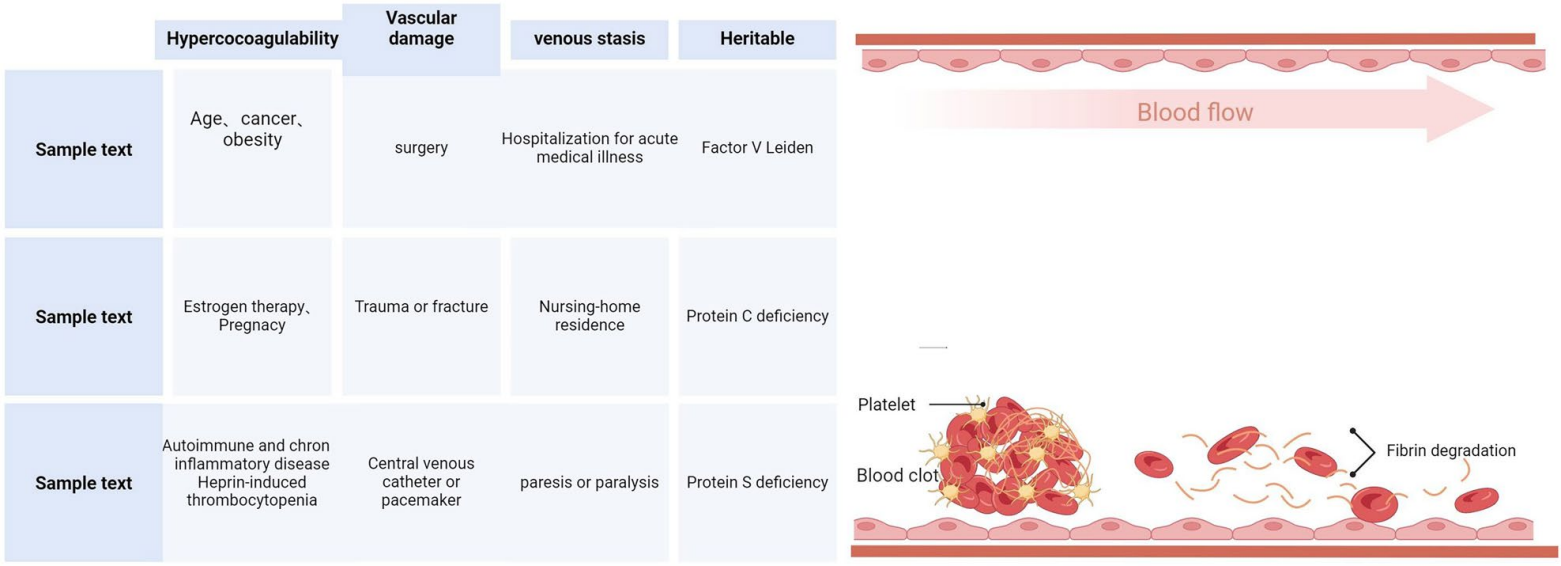
The process of venous thrombosis and the risk factors for DVT.

Most nontraumatic deep vein thrombosis originates primarily in the valves and sinuses of the calf veins [18–20]. In contrast to arterial thrombosis, the composition of venous thrombosis generally contains a large number of red blood cells and is not associated with endothelial denudation [21]. The site of occurrence and the pathologic composition fully suggest that the mechanism of venous thrombosis differs significantly from that of arterial thrombosis. As shown in Figure 1, the traditional view of venous thrombosis is that activated platelets and monocytes bind to tissue factor (TF) on the endothelial cell membrane and then to coagulation factor VIIa, triggering a chain reaction of coagulase enzymes that results in the formation of a fibrin clot [22]. However, in the last decade, as the study of thrombosis has advanced, it has been recognized that there is a very close association between inflammation and thrombosis; for example, patients with neocoronavirus pneumonia who progress to respiratory failure are at very high risk of thrombosis [23–25]. In addition, it has been demonstrated that autoimmune diseases such as antiphospholipid syndrome, inflammatory myositis, polymyositis and dermatomyositis, rheumatoid arthritis, sarcoidosis, Sjogren’s syndrome, autoimmune hemolytic anemia, systemic lupus erythematosus, systemic sclerosis, vasculitis, and inflammatory bowel disease contain specific inflammatory pathophysiological mechanisms, leading to the risk of arterial and venous thrombosis [26].

**Figure 2.**
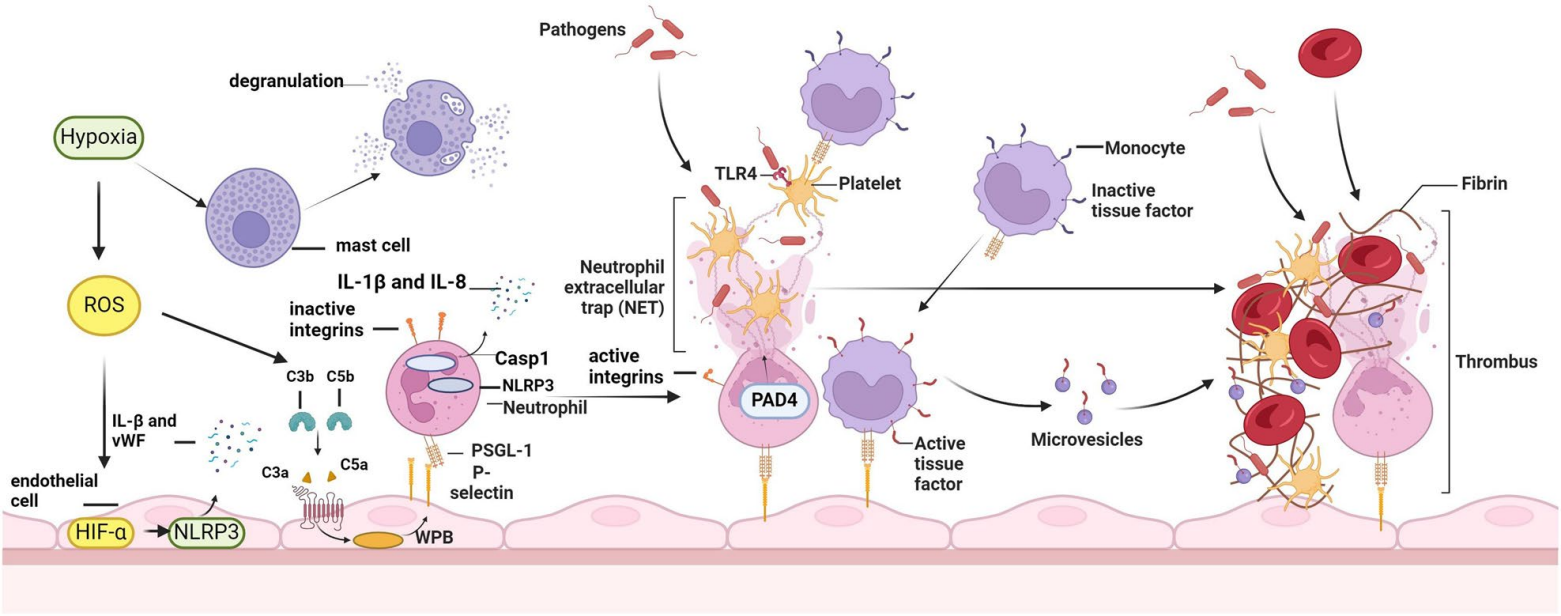
The propagation of immunothrombosis by leukocytes and platelets. Under hypoxic conditions, ROS leads to an increase in HIF-1α, which in turn induces the expression of NLRP3 inflammatory vesicles in endothelial cells. This leads to the secretion of IL-1β and promotes platelet activation and thrombosis. ROS also leads to mast cell activation, histamine release and complement activation. These substances induce the release of vWF and P-selectin-containing WPB vesicles from endothelial cells, followed by the aggregation of natural immune cells and platelets on the endothelial surface. The combination of HMGB1 released from platelets, TLR2 from monocytes and CXCR2 from neutrophils induces the release of NETs, a process regulated mainly by the PAD4 enzyme. In addition, the release of tissue factor by monocytes activates the exogenous coagulation cascade. TLR2: Toll-like receptor 2; CXCR2: chemokine receptor 2; PSGL-1: P-selectin glycoprotein ligand 1; ROS: reactive oxygen species; HIF-1α: hypoxia inducible factor-1; IL: interleukin; vWF: von Willebrand Factor; WPB: Weibel-Palade body; HMGB: high-mobility group protein; NETs: neutrophil extracellular traps; PAD4: peptidylarginine deiminase 4.

More interestingly, inflammation seems to be involved in the whole process of venous thrombosis (Figure 2). Venous thrombosis is mainly formed in veins with low shear, and it has been demonstrated that low shear can trigger the recruitment of immune cells through the upregulation of the NF-κB pathway [27]. The recruited immune cells could deliver procoagulant factors that initiate the coagulation cascade, such as TF released by monocytes, activating the coagulation system in VTE formation [22]. In addition, the stagnant blood flow in veins could lead to the secretion of inflammatory factors by mast cells as well as endothelial cells (histamine, etc.), and this could also promote thrombosis [28].

### Red blood cells

In clinical practice, erythrocytes were found to have a certain effect on coagulation, for example, the bleeding time of patients with anemia is prolonged even under normal platelet count conditions, and patients with true erythrocytosis have a certain risk of thrombosis [29,30]. However, the mechanisms by which erythrocytes alter coagulation are still unknown, and the effect on coagulation is currently attributed only to their effect on the rheology of the blood. Increasing of erythrocyte concentration leads to an increase in blood viscosity and thus promotes thrombosis [31,32]. Unlike normal double-sided depressed erythrocytes, in sickle cell disease, hemolytic anemia, and diabetes mellitus, erythrocytes with harder and poorly formed structure could promote platelets to free themselves from the edges of the vessels and finally promote thrombosis [33,34]. Additionally, when erythrocytes are stimulated by inflammatory factors, erythrocytes could produce relevant inflammatory particles [34,35], promoting the transfer of free heme to the vascular endothelium through the expression of tissue factor as well as phosphatidylserine, and the activation of thrombin-dependent complement, it then amplifies systemic inflammation, enhances the action of thrombin, and promotes the formation of blood clots [36]. Normal erythrocytes could display antithrombotic properties under certain conditions, for example, deoxygenated hemoglobin stimulates the release of NO from hemoglobin cysteine β93, leading to dilation of capillaries and reduced platelet reactivity [37].

### White blood cells

Patients with systemic infections as well as multiple organ failure, the interaction between platelets and immune cells activates the coagulation system as well as the complement system to promote thrombosis, resulting in microvascular obstruction and the absence of blood supply to the tissues [17]. It has been suggested that the immune cells involved in venous thrombosis are mainly neutrophils, mast cells, etc. [38], and that neutrophils perform a variety of cellular functions during venous thrombosis, including oxidative bursting, phagocytosis, degranulation, secretion of a variety of inflammatory factors, and the formation of extracellular traps for neutrophils [39]. It has been demonstrated that the primary metabolic pathway of neutrophils is glycolysis, but under conditions of low glucose availability, neutrophils could activate fatty acids for compensatory metabolism and utilize glutamate dehydrogenase to produce α-ketoglutarate to energize the TCA cycle [40,41]. Due to the low oxygen content of venous thrombi, neutrophils inhibit apoptosis by producing a number of glycolytic enzymes, including GAPDH, to produce ATP to maintain neutrophil survival and function [42,43].

### Platelet and platelet microvesicles

Platelets are important circulating cells, which are required to interact with inflammatory cells, and endothelial cells during thrombus formation [17]. The metabolism of activated platelets includes process of mitochondrial depolarization, ROS-dependent signaling, extensive cytoskeletal reorganization, and rapid protein synthesis [44–47]. In the setting of venous thrombosis, platelets are limited by nutrient as well as oxygen supply. Well-coupled mitochondria in platelets promote to produce ATP through glycolysis and oxidative phosphorylation [48]. In resting platelets, nearly 60% of the ATP comes from glycolysis, and the remaining 40% comes from oxidative phosphorylation [49]. When platelets are activated, the glucose transporter protein GLUT3 is overexpressed on the surface of the platelets, which enhances the uptake of glucose [50]. It has been demonstrated that the metabolism of activated platelets favors glycolysis over oxidative phosphorylation [48], which seems to correlate with oxygen- and nutrient-poor conditions in thrombi.

PMV(platelet microvesicles) is an extracellular vesicle differentiated from activated platelet membranes [51], which can mediate cell signaling by carrying cellular metabolites, lipids, proteins, and nucleic acids between cells and organs, and thus represents a new mode of intercellular communication in metabolic regulation [52,53]. Studies have now demonstrated that PMV is highly procoagulant, and more interestingly, under some pathological conditions, PMV can express tissue factor which in turn triggers blood coagulation [54], and there are already clinical data suggesting that a high percentage of PMV in plasma is a risk marker for future VTE [55]. The plasma levels of PMV are significantly higher in glucose-tolerant and diabetic patients, whose blood glucose may have a role in promoting the generation of PMV [56], PMV can promote the formation of venous thrombosis due to the presence of CD41, CD42b, and phosphatidylserine [57], and in addition, PMV can promote the production of inflammatory factors, such as IL-1, IL-6, and IL-8, which can in turn promote the formation of thrombi [58]. In addition to blood glucose can affect PMV, ROS can also have some effect on PMV, for example, nitric oxide synthase-2 and NADPH oxidase in neutrophils can promote the production of ROS, which in turn promotes the production of PMV [59].

## Mechanisms of influence of the metabolic microenvironment on DVT

**Figure 3.**
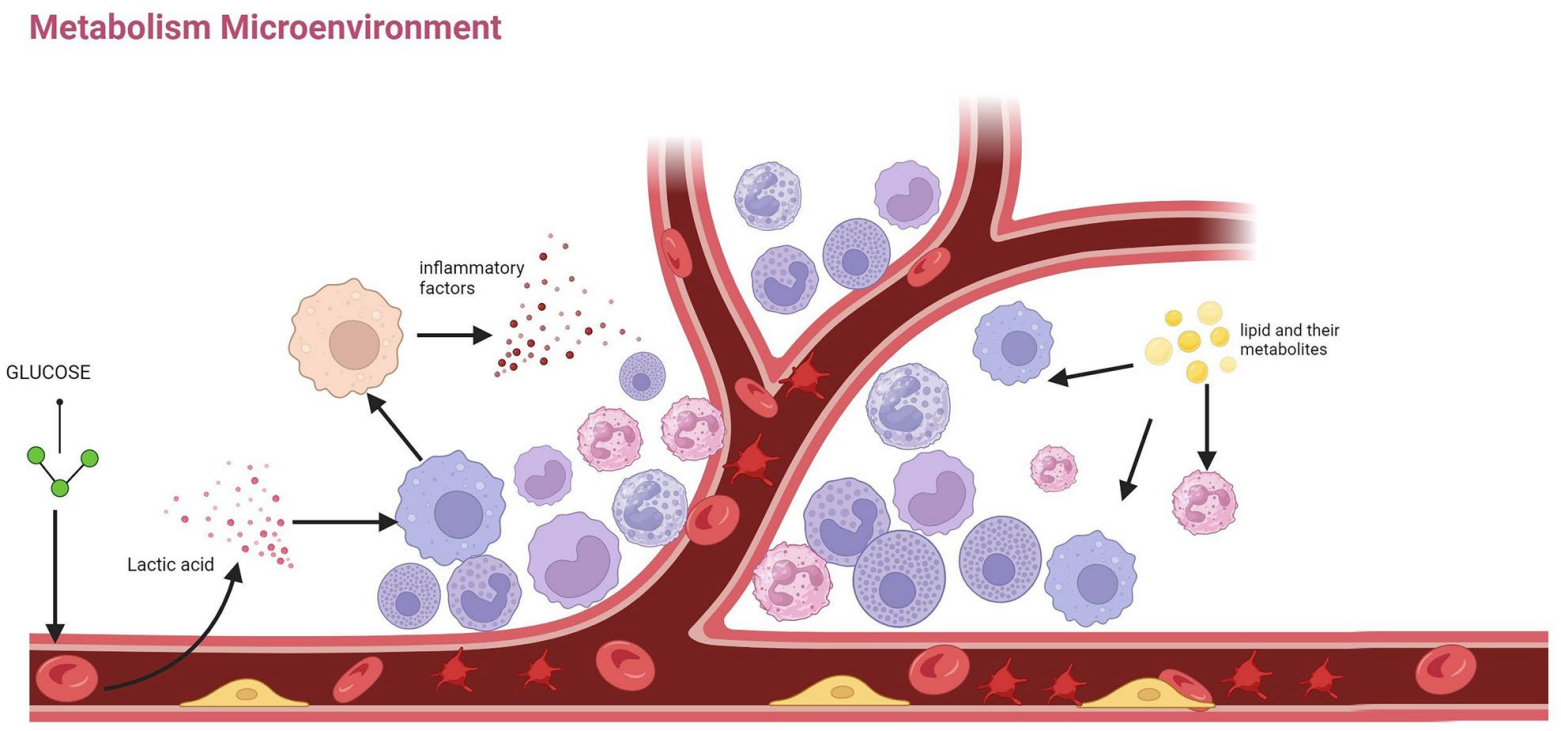
Erythrocytes and their cells metabolize sugars and secrete lactic acid and other acidic substances, resulting in an acidic microenvironment. After converting macrophages from the M1 type to the M2 type, M2-type macrophages secrete various inflammatory factors (IL-1, IL-8, and TNF-α) that promote the aggregation of immune cells and thrombus formation. Lipids and metabolites affect the fluidity of immune cells and platelet membranes, exacerbating their aggregation and activating their surface receptors (TLR and PPAR receptors). This leads to the recognition of immune cells by platelets and further promotes thrombus formation. IL: interleukin; TNF: tumor necrosis factor; TLR: Toll-like receptor; PPAR: peroxisome proliferator-activated receptor.

The identification of risk factors for venous thrombosis is essential for thromboprophylaxis in individuals. As shown in Figure 1, risk factors have been stratified Numerous studies have suggested that lipoproteins, blood glucose levels, and homocysteine are potential risk factors for VTE [60]. The metabolism of these substances is closely linked to inflammation (Figure 3), and we outline below the relationship between the metabolism of these types of substances and thrombosis.

### Glucose metabolism

There have been many studies describing age, obesity, and smoking as risk factors for DVT, with little mention of blood glucose [6]. However, in a study in Taiwan comparing 4967 patients with type 1 diabetes and 19,868 normal people, at a mean follow-up of 8.61 years, 0.3% of patients in the control group developed VTE, while 1.6% of patients in the diabetes group developed VTE [61]. In a recent clinical trial, it was found that the risk of pulmonary embolism was increased in patients undergoing hip and knee arthroplasty with preoperative blood glucose levels exceed 11.1 mmol/l [62]. Since the origin of DVT is usually in the deepest depression of the valve sinus at the valve site [21], hypoxia and high blood viscosity in this area are likely to induce changes in platelet and red blood cell glucose metabolism, which in turn promotes thrombosis.

The metabolites of thrombi are derived from erythrocytes, platelets and leukocytes in that order, so the metabolism of erythrocytes is likely to have a major impact on thrombosis. Generally, the tricarboxylic acid cycle process in glycolysis is the main way for cells to obtain energy. However, the cells involved in thrombosis do not receive an adequate supply of oxygen; therefore, cells in thrombi may not be able to carry out normal glucose metabolism [63]. It has been demonstrated that erythrocyte metabolism under hypoxic conditions is mainly dependent on competition between deoxyhemoglobin and glycolytic enzymes for the structural domain of band III cytoplasm [64], which is the main transmembrane protein of erythrocytes and has a strong affinity for deoxyhemoglobin and glycolytic enzymes (phosphofructokinase, lactate dehydrogenase, aldolase, etc.). Thus, it can inhibit the glycolytic pathway [64,65], resulting in an increase in lactate levels. In addition, thrombin-stimulated platelets could produce lactate [66], which is the most abundant metabolite in blood clots. Lactic acid itself could enhance blood coagulation and shorten whole blood clotting time in vivo and in vitro [67]. The acidic microenvironment caused by lactic acid also plays an important role in venous thrombosis. For example, macrophages can polarize in the acidic microenvironment and shift from the M1 phenotype to the M2 phenotype [68–70], and M2 phenotype macrophages can promote angiogenesis and secrete a variety of cytokines, such as basic fibroblast growth factor (BFGF), vascular endothelial growth factor (VEGF), interleukin-1 (IL-1), interleukin-8 (IL-8), tumor necrosis factor (TNFα) and nitric oxide [71–73]. Among these, TNFα can generate nitric oxide in the vessel wall to reduce platelet activation. In addition, TNFα acts on tumor necrosis factor receptor 2 (TNFR2) receptors to promote thrombosis [74,75]. A reduction in the size of the thrombus could be found after injecting sufficient TNFα into WT mice [75,76]. This may be because TNFα could improve fibrinolysis and collagenolysis and promote thrombus dissolution.

Hyperglycemia itself could lead to a hypercoagulable state of the blood [77], as well as reduced fibrinogen survival [78], indirectly promoting thrombosis. In addition, temporary hyperglycemia was found to activate platelet activity by activating PKC in platelets [79]. In addition, insulin, which is associated with hyperglycemia, seems to be involved in platelet activation [80]. It could cause the phosphorylation of endothelial nitric oxide synthase (eNOS), regulating NO synthesis and release [81,82]. NO activates guanylate cyclase through branched diffusion, leading to increased cGMP. The latter promotes vasodilation and the inhibition of platelet activity [83]. In summary, hyperglycemia itself has a thrombosis-promoting effect. The next point that has attracted more attention in recent years is advanced glycation end products (AGEs), which are the products of excess sugar and protein binding, and the AGE/receptor for advanced glycation end products (RAGE) axis, which is closely associated with inflammation and oxidative stress [84]. In a recent study, RAGE inhibition significantly suppressed lipid peroxidation malondialdehyde (MDA) and ROS levels in glycosylated human serum albumin (M-HSA)-stimulated mice, as well as endothelin-1 (ET-1) levels. In a previous study, it was shown that increased ET-1 expression may be involved in microthrombosis in retinal veins, which may complement the possibility of thrombosis [85]. In addition, blocking the AGE/RAGE pathway downregulated ET-1, which in turn reduced the damage to human umbilical endothelial cells (HUVECs) [86].

### Lipid metabolism

Previous studies have confirmed that lipids have a relatively important effect on arterial thrombosis, mainly in terms of hemostasis and fibrinolysis in patients [87–89]. Studies in recent years have shown that lipid-induced changes in the coagulation system are not only found in arterial thrombosis but are also detectable in venous thrombosis [90]. The lipids that have attracted the most attention are triglycerides, lipoproteins and cholesterol. High levels of triglycerides not only increase blood stasis [91] but also promote venous thrombosis by inhibiting tissue fibrinogen activator activity and increasing fibrinogen activator inhibitor-1 (PAI-1) with reduced fibrinolysis [92]. Lipoproteins also play a central role in the development and progression of venous thrombosis [93]. Low-density lipoprotein (LDL) is a complex lipoprotein containing different lipids, including triglycerides, phospholipids, and free cholesterol [94]. Oxidized LDL (OxLDL) affects thrombosis in various ways. OxLDL activates platelets by recognizing cluster of differentiation 36 (CD36) on platelets, and then, activated platelets exacerbate lipoprotein oxidation, further inducing thrombotic inflammation [95,96]. OxLDL can also induce platelets to form aggregates with monocytes, resulting in thrombosis [97]. Oxidized cholesteryl esters present in OxLDL could induce monocyte adhesion on top of endothelial cells and promote blood coagulation [98]. Oxidized phospholipids (OxPLs) isolated from the least oxidized LDL could stimulate endothelial cells to express B1 integrins as well as P-selectin, IL-8, and vascular endothelial growth factor [99–101] to induce monocyte recruitment and adhesion at the endothelium. OxLDL could upregulate the expression of the CD11b-CD18 adhesion receptor complex on neutrophil membranes and thus mediate neutrophil adhesion and migration [102]. Lysophosphatidylcholine in OxLDL has been shown to induce extracellular traps in neutrophils and promote thrombus formation [103]. Moreover, OxLDL could delay blood clotting by inhibiting the activity of coagulation factors VIII, IX and XI [104,105]. It has also been shown that OxLDL and OxPL could increase prothrombin activity and thrombin production [106,107]. The current inhibitory effect of oxidized lipoproteins on blood clotting may be weaker than their effective activation of vascular cells.

In some relevant Mendelian randomization trials, no significant causal relationship was found between the three classical lipids (LDL, HDL, and total cholesterol) and VTE, whereas there was an association between VTE and fatty acids [108]. Fatty acids and metabolites can affect different immune cell populations, including neutrophils and macrophages, by regulating the fluidity of cell membranes [109]. In addition, they are able to activate relevant recognition receptors on the cell membrane, such as Toll-like receptors (TLRs) and peroxisome proliferator-activated receptors (PPARs), in the nuclear receptor family [109]. Neutrophils recognize platelets through TLR receptors and trigger the formation of neutrophil extracellular traps [110,111], leading to thrombosis. PPAR contains three isoforms: PPARα, PPARβ, and PPARγ. They are highly expressed in oxidative tissues and regulate oxidative phosphorylation and substrate oxidation [112–114]. In addition, PPAR has a wide range of natural ligands, including fatty acids, acyl coenzyme A and glycerophospholipids [115,116], making it a very important regulator of lipid metabolism. Hyperlipidemia promotes high expression of PPARα in platelets and mediates platelet activation and thus thrombus formation by regulating downstream p38 and Akt to promote the secretion of mitogenic bodies [117]. High-density lipoprotein (HDL) could inhibit thrombosis by affecting exogenous coagulation pathways and fibrinolysis and reducing blood viscosity [118]. HDL can also inhibit tissue factor and inactivate coagulation factors Va and VIIIa in the presence of activated protein C [119]. Moreover, docosahexaenoic acid (DHA) has anti-inflammatory effects by reducing nuclear factor κB (NF-κB) in peripheral blood mononuclear cells and TNFα mRNA expression in peripheral blood mononuclear cells [120].

### Hypoxic conditions and oxidative stress

Current epidemiological statistics show a significant increase in the incidence of deep vein thrombosis and pulmonary embolism in populations at high altitudes [121–123]. Several studies have demonstrated that the hypoxic response could lead to platelet, endothelial and lymphocyte activation and thus promote inflammatory thrombosis [124,125]. Significantly elevated levels of NLRP3, interleukin (IL)-1β, IL-18 and cystatin-1 in peripheral blood could be observed from environments where DVT occurs at high altitude [126–128]. Hypoxia induces high expression levels of IL-1β in platelets [110,129–131] and upregulates IL-1β and NLRP3 in monocytes [126,132]. IL-1β can induce platelet activation and enhance platelet hyperresponsiveness, promoting platelet aggregation [133]. Activation of NLRP3 inflammatory vesicles could trigger aeroderm D-dependent lytic pyroptosis in macrophages, which in turn leads to the secretion of tissue factor by macrophages and promotes thrombosis [134]. In addition, hypoxia has also been shown to increase Ca21 in the platelet cytoplasm and thus activate calpain in the platelet membrane to promote thrombosis [135,136].

Age, an independent risk factor for VTE, is often associated with oxidative stress [137,138], as aging is characterized by excessive production of reactive oxygen species (ROS) [139]. ROS promote venous thrombosis by increasing the expression of tissue factor in endothelial cells and monocytes, activating platelets, and inducing thrombotic inflammation [140]. Erythrocytes are a major source of ROS, and they release large amounts of ROS through the activation of NADPH oxidase and oxidation of hemoglobin [141]. ROS could facilitate the influx of calcium ions by activating calcium channels that not only disrupt the erythrocyte membrane but also expose phosphatidylserine on the erythrocyte membrane, which further activates thrombinogen [142,143]. Erythrocyte membrane damage leads to the release of free hemoglobin and heme, which further activates hypoxia-inducible factor (HIF) production *via* the TOLL-like receptor signaling pathway. This further induces inflammation and increases endothelial permeability [144]. When this redox heme is transferred to endothelial cells, it can trigger H2O2-mediated endothelial damage, stimulating neutrophil recruitment and the formation of NETs and NLRP3 inflammatory vesicles [145]. In addition, ROS can oxidize arachidonic acid to activate platelets and affect the expression of P-selectin and soluble CD40L on the platelet surface to promote platelet–leukocyte interactions [146–148]. It has been shown that ROS could also induce the expression of adhesion molecules on immune cells to promote the recruitment and activation of immune cells [149], and it could activate calcium channels on mast cells. This leads to an increase in the concentration of calcium ions in the mast cell cytoplasm and promotes mast cell degranulation [150]. In addition, a link between mast cell degranulation and venous thrombosis has been previously demonstrated [28].

### Homocysteine metabolism

The hypothesis that hyperhomocysteinemia is a causal risk factor for venous thrombosis is currently speculated in the results of a number of ‘Mendelian randomization’ trials [151]. Homocysteine is one of the more important amino acids in the body. When various factors induce the accumulation of homocysteine in the blood, excessive homocysteine results in cell, tissue, and organ damage [152]. Additionally, current research on HCY demonstrates that HCY can induce the expression of vascular cell adhesion molecule-1 (VCAM-1) in endothelial cells to enhance the adhesion of monocytes to endothelial cells [153,154]. HCY could also disrupt tissue mitochondrial singlet transport chain complex I. This leads to damage to the electron transport cascade, which in turn promotes the production of ROS in mitochondria. This process causes damage to cellular tissues and inflammation [155]. Therefore, clinically, homocysteine is an effective independent risk factor for degenerative diseases such as cardiovascular disease, chronic kidney disease and diabetes [156]. The metabolic process is divided into two categories: 1) HCY is converted to methionine by remethylation; and 2) HCY is converted to cysteine by the action of cystathionase condensing enzyme and cystathionase [157]. Methionine metabolism is involved in many cellular functions, including methylation reactions, redox maintenance, and folate metabolism [158]. Some studies suggest that methionine obtained in the diet could have a significant impact on cellular metabolism [158]. However, few studies on methionine metabolism and venous thrombosis are still lacking.

## Therapeutic targets

The current targets of antiplatelet therapy are mainly platelet activating receptors as well as cyclooxygenase [159]. Compared to arterial thrombosis, platelet activation is weaker in venous thrombosis [160]; therefore, the therapeutic effect of antiplatelet aggregation pharmacotherapy for venous thrombosis is not effective. However, on the other hand, platelet metabolism has an impact on thrombus formation. For example, platelet glycolysis could drive NADPH synthesis via the pentose phosphate pathway, leading to ROS production [161] and thrombogenesis. Pyruvate dehydrogenase kinase (PDK) and pyruvate kinase M2 (PKM2) are key enzymes in platelet glycolysis [162], but there is no concrete evidence to prove whether they have a certain effect on venous thrombus formation. Although the cornerstone of current treatment for venous thrombosis is anticoagulation, the risk of bleeding is not negligible [15]. Thus, anti-inflammation seems to hold great promise in the field of treating venous thrombosis. There are already studies that demonstrate feasibility. Such studies reported the use of deoxyribonuclease I (DNaseI) to inhibit venous thrombosis by pharmacologically disrupting NETs [163] and inhibiting receptors recognized by high mobility group protein-1 (HMGB1), including RAGE, TLR2, TLR4, and other receptors to inhibit venous thrombosis [164]. These inflammatory targets are highly associated with the metabolic microenvironment. For example, it has been shown that hyperglycemia could increase the release of NETs in patients with type II diabetes [165]. In addition, venous thrombosis can lead to unstable circulation and inadequate perfusion, so tissues are susceptible to ischemia, hypoxia, and consequently oxidative stress [166], so some specific markers of oxidative stress can be used as markers in the diagnosis of venous thrombosis as well as in the treatment of venous thrombosis. It has been demonstrated that malondialdehyde and reactive oxygen species levels are increased in patients with venous thrombosis [166], and that these substances are involved in the oxidative stress response. In addition, asymmetric dimethylarginine (ADMA), an amino acid commonly found in tissues that causes vasoconstriction and damage to endothelial cells [167], has been shown to be significantly elevated in patients with PTE [168]. Elevated levels of myeloperoxidase (MPO), a member of the heme peroxidase superfamily, are associated with increased inflammation and oxidative stress, and in patients with venous thrombosis, elevated levels are often associated with poor prognosis [169].

Activated neutrophils are responsible for promoting venous thrombosis through multiple mechanisms, and the three most prominent mechanisms are the production of tissue factors, the release of neutrophil extracellular traps, and the activation of inflammatory vesicles [170]. NETs are composed of nuclear DNA, histones, and neutrophil nuclear-derived granule proteins. NET production and release are mainly influenced by two enzymes, namely, nicotinamide adenine dinucleotide phosphate (NADPH) oxidase and nuclear peptidylarginine deaminase 4 (PAD4) [171,172]. Guanylated histone H3 in NETs can interact with vascular hemophilia factor (vWF) to promote thrombosis [173]. In addition to activating coagulation factor XII to activate the coagulation cascade to enhance venous thrombosis [174], NETs have now been shown to hydrolyze tissue factor pathway inhibitors to indirectly promote thrombin and fibrin clot production [175].

Glucose is a metabolic substrate and a major source of energy for neutrophils, which express the specific glucose transport proteins glucose transporter protein 1 (GLUT1) and glucose transporter protein-4 (GLUT4) on their cell membrane surface [176,177]. However, compared to other cells, neutrophils have fewer mitochondria, and they rarely participate in adenosine triphosphate synthesis [178]. Thus, the formation of NETs is mainly dependent on glycolysis. Therefore, glucose metabolism has a certain influence on the synthesis and release of neutrophils and NETs. For example, glucose overload and deficiency could affect the chemotaxis and phagocytosis of neutrophils [179]. In addition, it has been suggested that the release of NETs is strictly dependent on exogenous glucose and glycolysis. Therefore, the formation of NETs could be completely inhibited by using glycolysis inhibitors [41]. Oxidative phosphorylation and pentose phosphate pathways are not the main energy sources of NETs, but they function as secondary messengers to regulate the synthesis of NETs [180–182]. For example, the purinergic receptor P2X1 acts as a relevant signaling pathway for NETs [183]. The pentose phosphate pathway is involved in the production of NADPH oxidase-dependent ROS, which indirectly promotes the formation of NETs [184]. However, studies on whether fatty acids and other metabolites affect the synthesis and release of NETs are lacking. Neutrophils are the main source of ROS, and oxidative stress in NETs is highly associated with ROS. For NETs in the NADPH enzyme-dependent pathway, it has been shown that NADPH oxidase or ROS scavengers could inhibit NET formation. However, experimental data on whether they have an effect on venous thrombosis are not sufficient [185].

## Conclusions

The cellular metabolism involved in venous thrombosis is often accompanied by the pathological processes of oxidative stress and cellular autophagy, which are often associated with inflammation. ROS have been shown to promote the release of inflammatory factors and the synthesis and release of neutrophil extracellular traps that promote thrombosis. Blood glucose concentrations can cause platelet activation and modulation of NO secretion in vascular endothelial cells, intermittently promoting thrombosis. In addition, glucose, as a major substrate of cellular metabolism, is metabolized by pathways including oxidative phosphorylation, the pentose phosphate pathway, and glycolysis. These pathways can indirectly affect venous thrombosis by affecting neutrophils and other cells as secondary messengers and receptor ligands. Additionally, these metabolites (ATP, lactate, pyruvate) not only lead to the production of inflammatory factors, such as tumor necrosis factor-α (TNF-α), by immune cells to indirectly affect thrombosis but also increase platelet activity, synthesis and release of extraneutrophil traps and vasoconstriction. Lipids and corresponding metabolites can reduce blood flow and alter platelet membrane fluidity, leading to blood stasis. Moreover, lipid metabolism in macrophages has been shown to be associated with inflammation and the release of inflammatory factors involved in venous thrombosis. Several anti-inflammatory strategies have been proposed for venous thrombosis, and one of the more intriguing strategies is the neutrophil extracellular trap. Glucose is an important source of energy for cells and has a major impact on the formation of NETs. ROS produced by cellular metabolism can also influence the formation of NETs. Therefore, based on these theories, new developments in the field of nutrition, including intermittent fasting and CR diets, have recently been shown to have an important role in the prevention of thrombosis; however, when considering the translation of these interventions into clinical treatment, we need more research on metabolism and venous thrombosis.

## Data Availability

Data sharing is not applicable to this article as no new data were created or analyzed in this study.
